# Improving Drug Loading of Mucosal Solvent Cast Films Using a Combination of Hydrophilic Polymers with Amoxicillin and Paracetamol as Model Drugs

**DOI:** 10.1155/2013/198137

**Published:** 2013-06-11

**Authors:** Joshua Boateng, Justine Mani, Farnoosh Kianfar

**Affiliations:** Department of Pharmaceutical, Chemical and Environmental Sciences, Faculty of Engineering and Science, University of Greenwich at Medway, Central Avenue, Chatham Maritime, Kent ME4 4TB, UK

## Abstract

Solvent cast mucosal films with improved drug loading have been developed by combining carboxymethyl cellulose (CMC), sodium alginate (SA), and carrageenan (CAR) using paracetamol and amoxicillin as model drugs and glycerol (GLY) as plasticizer. Films were characterized using X-ray powder diffraction (XRPD), scanning electron microscopy (SEM), folding resilience, swelling capacity, mucoadhesivity, and drug dissolution studies. SA, CMC, and GLY (5 : 3 : 6) films showed maximum amoxicillin loading of 26.3% whilst CAR, CMC, and GLY (1 : 2 : 3) films had a maximum paracetamol loading of 40%. XRPD analysis showed different physical forms of the drugs depending on the amount loaded. Films containing 29.4% paracetamol and 26.3% amoxicillin showed molecular dispersion of the drugs while excess paracetamol was observed on the film surface when the maximum 40% was loaded. Work of adhesion was similar for blank films with slightly higher cohesiveness for CAR and CMC based films, but the differences were significant between paracetamol and amoxicillin containing films. The stickiness and cohesiveness for drug loaded films were generally similar with no significant differences. The maximum percentage cumulative drug release was 84.65% and 70.59% for paracetamol and amoxicillin, respectively, with anomalous case two transport mechanism involving both drug diffusion and polymer erosion.

## 1. Introduction 

The most widely used and preferred route of drug administration is the oral route, providing both safety and patient compliance [1, 2]. However, limitations such as hepatic first pass metabolism and enzymatic degradation of drugs, especially acid labile drugs such as proteins and peptides within the gastrointestinal tract, have led to the study of alternative routes for drug delivery [3–6]. In recent times, a great deal of interest has focused on the use of the mucosal lining of body cavities including the oral mucosa. The oral cavity has a large accessible surface area, thus bringing about sufficient drug absorption. Different regions within the oral cavity useful for effective drug delivery are buccal, sublingual, palatal, and gingival. The most widely used are buccal and sublingual mucosae due to lack of keratinised epithelium and they have been employed for both local and systemic drug delivery [7–9]. Buccal films (oral strips) are ultrathin strips with an active ingredient and other excipients. They are largely suitable for administration to paediatric and geriatric populations mainly due to their ease of administration and portability [10].

Furthermore, buccal films have distinct advantages over other oral formulations such as tablets and syrups. Whilst oral disintegrating tablets (ODTs) are fragile and brittle, requiring special packaging to protect during storage and transportation, films are flexible and less fragile. This facilitates ease of handling, transportation, and storage [11]. In comparison to drops or syrup formulations, each strip guarantees accuracy of the dose administered. Films are popular amongst patients having difficulty swallowing tablets and capsules (dysphagic patients). This is because the strips readily hydrate in the moist buccal cavity enabling the dosage form to be administered at any geographical location and any time depending on the convenience of the individual without the need for clean water [12, 13]. The drug can be directly absorbed into the systemic circulation through the highly vascular oral mucosa and therefore avoid hepatic first-pass metabolism which enables the administration of a lower dose, thus minimizing the adverse effects related to the drug molecule [12]. Factors governing the extent of drug absorbed from the mucosal surface include the concentration the active ingredient, the vehicle used for its delivery, contact time with the mucosal surface, venous drainage of the mucosal tissues, degree of ionization of the drug, pH at the site of absorption, and relative lipid solubility of the drug [14]. The performance of many drugs can be extensively enhanced by the use of bioadhesive polymers such as hydrogels that extend the contact time of the drugs with these tissues [15, 16].

Though the previous advantages have resulted in high patient compliance and improved therapy at a nominal cost [10], there are certain limitations. Drug loading into thin films in significant amounts can be very challenging as there is very little volume available for uniform drug distribution due to their thin nature. This can affect drug release characteristics and their cosmetic appeal to patients [17]. This paper discusses the preparation, development, optimization, and characterization of hydrogel-based films using varying combinations of carboxymethyl cellulose (CMC), carrageenan (CAR), and sodium alginate (SA) with glycerol (GLY) as the plasticizer. The aim is to increase the drug loading capacity of two model water soluble drugs, paracetamol and amoxicillin, which are normally loaded into tablets or capsules in relatively high amounts (500 and 250 mg per dose, resp.) and well characterised in terms of physical and chemical properties. 

## 2. Materials and Methods

### 2.1. Materials

Paracetamol was obtained from Acros Organics (Leicestershire, UK); *κ*-carrageenan (CAR) (Gelcarin NF 812) was a gift from BASF (Surrey, UK). General purpose grade sodium alginate (SA) was obtained from Fisher Scientific (Leicestershire, UK) while carboxymethyl cellulose (CMC) (low viscosity: 2% aqueous solution at 25°C, 400–800 cps) and amoxicillin were obtained from Sigma-Aldrich (Gillingham, Dorset, UK). Glycerol (GLY) having a density 1.26 was obtained from Fluka (Dorset, UK). 

### 2.2. Method Development

#### 2.2.1. Preparation of Gels and Films

Preliminary experiments were performed with the various polymers, individually and in combination to determine the minimum amount required to form aqueous gels that were clear with no lumps and easy to pour with no entrapped air bubbles. The method used was vortex hydration with heat [18]. Glycerol (GLY) was first dissolved in hot distilled water, heated to 70°C with constant stirring using a magnetic stirrer. The dry polymers were dispersed in small quantities into the vortex of the GLY solution. The mixture was stirred for one hour to enable the polymers to dissolve completely. Different polymer to GLY ratios were investigated to determine the effect on film characteristics including folding resilience, residual water, and mucoadhesion. The uniform gel obtained was covered with foil and kept undisturbed for 10 minutes to allow entrapped air to escape. Different quantities (20 g, 25 g, 30 g, and 35 g) of the gels were poured into Petri dishes (86 mm diameter) to determine the optimum amount required to form films having optimum thickness, which enabled easy removal from the Petri dish.  Table 1 shows the different polymer-plasticizer combinations used to prepare the optimum gels. The gels were dried for 24 hours in an oven using two different drying temperatures (45°C and 60°C). The dried films were carefully removed from the Petri dishes and visually examined and the thickness measured using a micrometer screw gauge and stored for further evaluation. 

#### 2.2.2. Drug Loading into Films

Two different approaches were used to load the maximum amount of drug that was uniformly distributed within the film whilst maintaining film transparency and homogeneity when observed visually.

(i) The gel was prepared as described earlier and allowed to cool to about 50°C. The drug (paracetamol/amoxicillin) was added to the gel and stirred further. The mixture was kept on a bench to allow removal of air bubbles, poured into Petri dish (86 mm diameter), and dried at 60°C for 24 hours to produce the film.
(ii) The drug was initially dissolved in the deionised water and the plasticizer and polymers were added to the resulting solution in small proportions to ensure uniform mixing. Upon obtaining a uniformly mixed gel, the air bubbles were allowed to escape and dried in Petri dishes (86 mm diameter), at 60°C for 24 hours [11].

### 2.3. Thermogravimetric Analysis (TGA)

TGA was employed to determine the residual water in the films. About 3–10 mg of sample was weighed, placed in T-zero aluminium pans (open), and heated from 25°C to 150°C at a heating rate of 10°C/min under a constant flow of dry nitrogen gas. The weight loss was determined by a high resolution TGA 2950 instrument (TA Instruments, Crawley, UK).

### 2.4. Folding Resilience

The mechanical properties of the films were investigated by measuring the folding resilience which was estimated manually for the blank and drug loaded films. A strip of film (3 × 3 cm) was cut evenly and repeatedly folded at the same place until it was torn. The folding resilience was determined by the number of times the film could be folded without tearing. 

### 2.5. X-Ray Powder Diffraction (XRPD)

XRPD was performed on polymer films containing 29.4% and 40% paracetamol as well as 26.3% amoxicillin using a D8 Advance (Bruker, Karlsruhe, Germany) instrument. The experiment was performed in transmission mode with 2-Theta scale 5–42°, an exit slit of 0.6 mm, step size 0.02°, and counting time of 0.3 sec/step.

### 2.6. Scanning Electron Microscopy (SEM)

Polymer films were fixed with the help of double-sided copper adhesive tape, gold coated, and analysed in a Stereoscan 360, Cambridge Instruments (Cambridge, UK). SEM Images of the gold-coated films were obtained at 10 kv intensity and magnification of ×500. 

### 2.7. Hydration and Swelling Studies

Hydration studies were performed to investigate the maximum time required for films to hydrate and swell to the maximum capacity in 30 mL phosphate buffer solution. The buffer solution was prepared from KH_2_PO_4_ and NaOH (0.1 M) to obtain a pH of 6.5 simulating salivary conditions. The films were cut to 3 cm × 3 cm square strips and immersed in the buffer solution. Samples were weighted initially before immersing in PBS solution and the weight change was measured every 20 minutes up to 120 minutes. The experiments were performed in triplicate.

### 2.8. Mucoadhesivity Studies

Mucoadhesivity studies were performed using a 75 mm (P/75) probe attached to a TA HD plus (Stable Micro System, UK) texture analyser equipped with a 5 kg load cell and Texture Exponent-32 software programme. The films were cut to 3 cm × 3 cm strips and attached to the surface of the probe by double sided adhesive tape. A Petri dish containing set agar gel, equilibrated with 200 *μ*l of buffer solution (pH = 6.5), simulating the pH conditions in the buccal environment, was used to represent the mucosal surface. The samples were placed on the agar surface for one minute to allow complete hydration and complete contact before detachment. The Texture Analyser was programmed to work in tension mode and the probe detached at a pretest speed of 0.5 mm/sec and test speed of 1 mm/sec. The maximum force required to separate the film specimen from the agar surface was determined. 

### 2.9. *In Vitro *Drug Dissolution Studies

Before dissolution studies, a calibration curve was plotted by measuring the absorbance of five different concentrations (30–50 *μ*g/mL) of paracetamol and amoxicillin using A Varian UV spectrophotometer. Drug release experiments were carried out on formulations containing 40% paracetamol and 26.3% amoxicillin using a previously developed method [11]. The films were cut into squares of sides 2 cm and immersed in 50 mL phosphate buffer solution (pH 6.5) in a beaker. The temperature of the solution was maintained at 37°C and was constantly stirred at a speed of 40 rev/min using a magnetic stirrer. Samples were withdrawn at 5 minutes intervals for 100 minutes and replaced with fresh dissolution medium on each occasion, till the entire film dissolved. The time interval between sample withdrawal was increased to 10 minutes and then every 30 minutes. The final sample was withdrawn the next day, allowing the film to remain overnight under these conditions. The absorbance was determined after performing necessary dilutions at wavelengths of 242 nm and 272 nm for paracetamol and amoxicillin, respectively. The percentage cumulative drug release was calculated and plotted against time. The kinetics of paracetamol and amoxicillin release from the films was assessed by fitting the dissolution data (percentage release against time) to the Korsmeyer-Peppas equation in order to determine the release mechanism of both drugs from the optimised formulations.

## 3. Results and Discussion

### 3.1. Formulation Development

Different polymers and GLY (plasticiser) were used in various combinations for the formulation of mucoadhesive films with the aim of achieving improved drug loading. GLY was chosen as plasticiser as it has been shown previously to be a suitable plasticiser for hydrophilic polymers such as CMC and SA [18]. The first step involved the optimization of the gel and film formation process. For gels prepared by the first approach described earlier, the drug precipitated by recrystallisation during the film drying process as water evaporated from the gel. In the case of the second approach, the film obtained showed uniform drug distribution with no crystal aggregates visible to the naked eye. Therefore, the second approach where drug was first dissolved in the deionised water before addition of the plasticizer and polymers was the method of choice for preparing all subsequent gels. This seems to be related to the proper uniform mixing of the drug in solution before gel formation in the second approach, compared to the first approach where addition of the drug to the viscous gel solution could retard proper uniform distribution of the drug within the gel formulation mixture. We have shown in previous reports that initially dissolving paracetamol in hot distilled water before addition of the plasticiser and polymer to form the gel produced more uniform and homogeneous films [19].

It was found that combination of polymers yielded better films compared to single polymers when plasticised with GLY. The three polymers in question are all hydrophilic polymers with several OH groups available for interacting with the GLY, which acts by reducing the interchain interactions and ultimately increasing the specific volume with a resultant decrease in glass transition [18]. For single polymers, relatively high amounts of GLY generally result in excessive plasticization with the formation of weak and sticky films which are not ideal.

**Figure 1:**
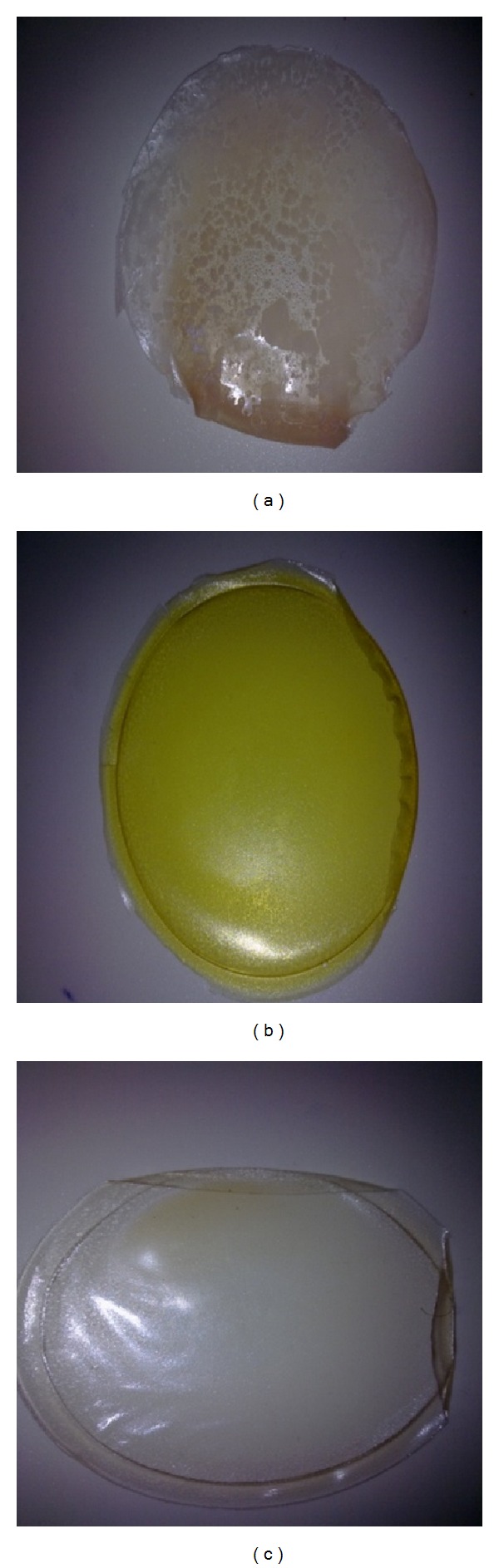
Representative digital images showing (a) typically rejected patchy nonhomogeneous film, (b) optimised amoxicillin loaded film, and (c) optimised paracetamol loaded film.

 Combination of two or more polymers seems to reduce this effect, possibly by reducing plasticising effect of the GLY. It has also been reported by Pawar and coworkers that blending of hydrophilic polymers such as CAR and SA with the naturally sticky polymer polyox improved the physical properties compared to films obtained from only polyox [20]. Therefore, films comprising more than one polymer were used for drug loading. Figures 1(a)–1(c) show typical digital images of films that were deemed either ideal (selected for further work) or nonideal (rejected). 

Petri dishes filled with 20 g and 25 g of the gel produced films that were not easily recovered without damaging the film due to either being too brittle or too thin. On the other hand, the film prepared by pouring 35 g of gel was too thick making it opaque and nonuniform. The ideal film (readily removed without being damaged and with sufficient transparency) was obtained using 30 g of gel. As a result, all subsequent films were prepared by pouring 30 g of gel. The thickness for these films ranged between 0.3 and 0.4 mm. The gels kept at 45°C for 24 hours did not dry sufficiently to form films while drying at 60°C for 24 hours produced films which were completely dried and easy to remove and was the drying temperature of choice for all subsequent films. The plasticized gels and their corresponding films were evaluated for characteristics such as ease of pouring, transparency, appearance, thickness, brittleness, and stickiness [21]. 

### 3.2. Drug Loading

The formulations with the previous ideal characteristics were selected for drug loading as shown in Table 1. Films formulated with gels combining CAR : CMC : GLY (1 : 2 : 3) showed the maximum drug loading of 40% (of total dry weight) for paracetamol whilst those comprising SA : CMC : GLY (5 : 3 : 6) yielded films with a maximum amoxicillin loading of 26.3%. This is interesting as we have previously showed a maximum paracetamol loading of 12.7% (of total dry weight) in CAR films modified with poloxamer and plasticised with PEG 600 [11]. These results seem to indicate the advantage of combining different hydrophilic polymers to help increase the drug loading capacity of solvent cast films. These films with ideal optical appearance (transparent with no drug recrystallization visible to the eye) were selected for further development and physical characterisation. 

### 3.3. Thermogravimetric Analysis (TGA)

According to Table 2, the blank film comprising CAR : CMC : GLY had significantly higher residual water (8.18%) compared to films containing SA : CMC : GLY with water content of 2.44%. Similar observations were made for the drug loaded films though the difference in water content was not significant as observed between the blank films. This seems to indicate that CAR has higher water sorption characteristics compared to SA. The overall implication of these results is that films comprising CAR : CMC : GLY are expected to be more flexible which was observed visually through handling though no tensile tests were performed. This could be attributed to the known plasticising effect of water which is additive to that of GLY [18]. 

### 3.4. Folding Resilience

Both blank and drug loaded films were folded repeatedly on the same line to determine their flexibility and resistance to deformation. Results for films comprising CAR : CMC : GLY (1 : 2 : 3) with or without drug demonstrated significant resilience as they remained intact after twenty folds. The film containing SA : CMC : GLY (5 : 3 : 6), however, showed significantly lower resilience tearing after only three folds. This could be attributed to the differences in proportion of polymer compared to plasticiser but also to the residual water content as noted earlier. Generally, the resistance of a film to deformation (tensile strength) is determined by the amount and type of polymer used and by the content of plasticiser. The latter formulation with lower water content is expected to be less plasticised and therefore more brittle, hence, exhibiting greater potential for tearing along lines of weakness. 

### 3.5. X-Ray Powder Diffraction (XRPD)

XRPD studies of the films were performed in order to determine the physical form (crystalline or amorphous) of the drug incorporated within the film. 

**Figure 2:**
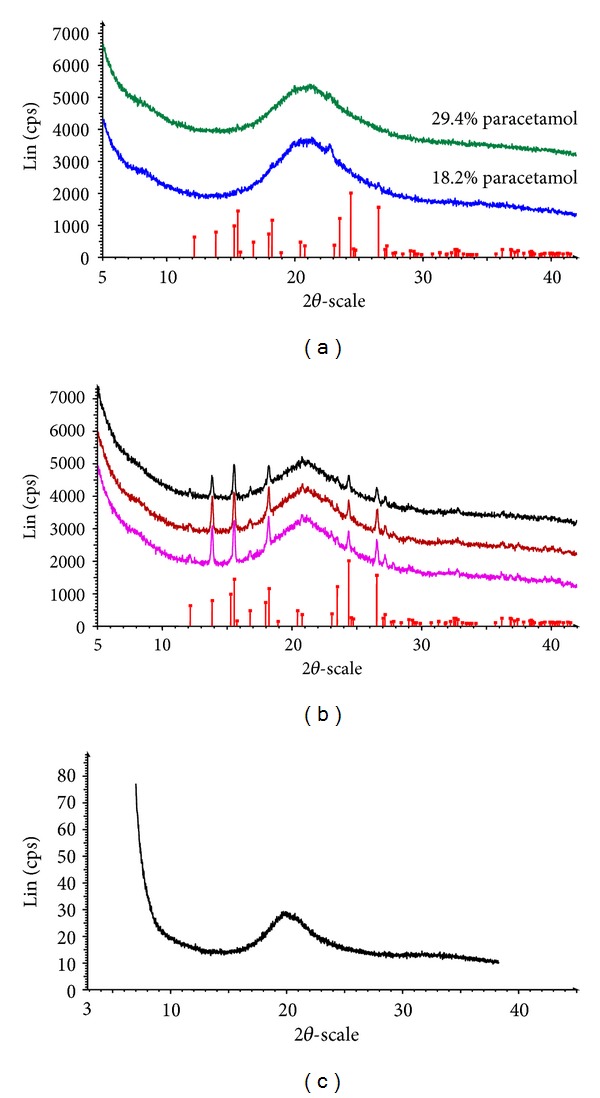
XRPD patterns of CAR : CMC : GLY (1 : 2 : 3) films containing (a) 29.4% and 18.2% paracetamol (b) 40% paracetamol, and (c) SA : CMC : GLY (5 : 3 : 6) films containing 26.3% amoxicillin. The drug contents have been calculated as a percentage of total dry weight of the films.

XRPD analysis of different films, indicated varying results depending on the percentage drug loading.  Figure 2(a) is the XRPD pattern for films containing 29.4% paracetamol, which clearly indicates that the drug was amorphous or molecularly dispersed within the film matrix.  Figure 2(b) shows the XRPD pattern for films containing the highest amount (i.e., 40%) of paracetamol. The main peaks for paracetamol, in particular the expected peak at 2*θ* of 24.5°, were clearly shown indicating the presence of excess recrystallised paracetamol on the film surface. This is very interesting and can give an indication of how much of the loaded drug was molecularly dispersed. However, this requires further studies to determine an exact loading of paracetamol at which recrystallization occurs.  Figure 2(c) shows the amorphous nature of amoxicillin in the film. 

### 3.6. Scanning Electron Microscopy (SEM)

**Figure 3:**
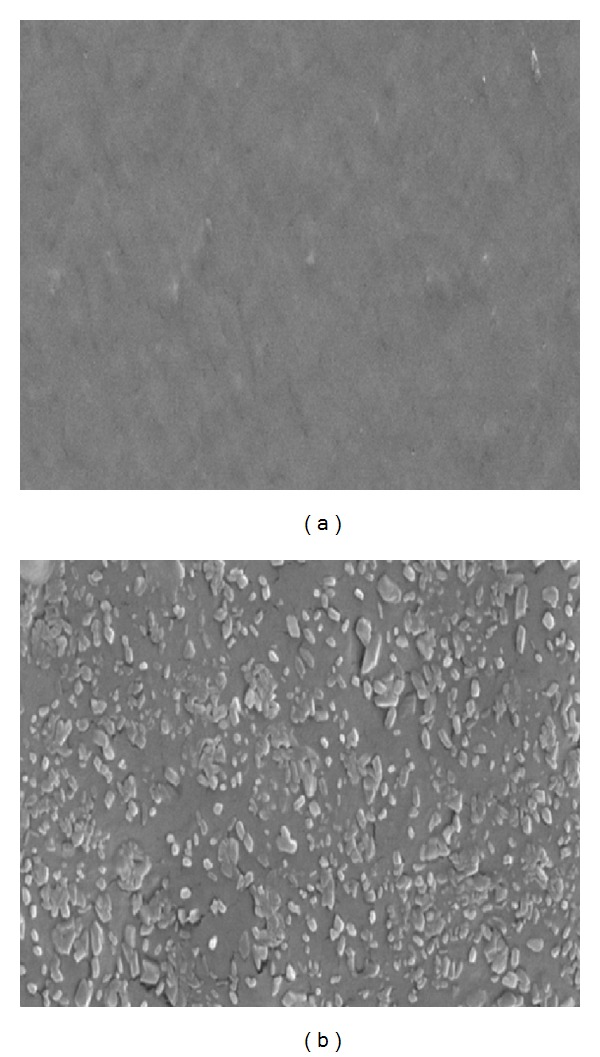
Representative SEM images of (a) blank CAR : CMC : GLY film and (b) the corresponding paracetamol (40%) loaded film.

Figure 3(a) shows blank films containing CAR : CMC : GLY, and Figure 3(b) shows the corresponding film loaded with 40% of paracetamol. The drug loaded film showed the excess of recrystallised drug clearly present on the film surface as suggested by the XRPD results. 

### 3.7. Hydration and Swelling Profiles

**Figure 4:**
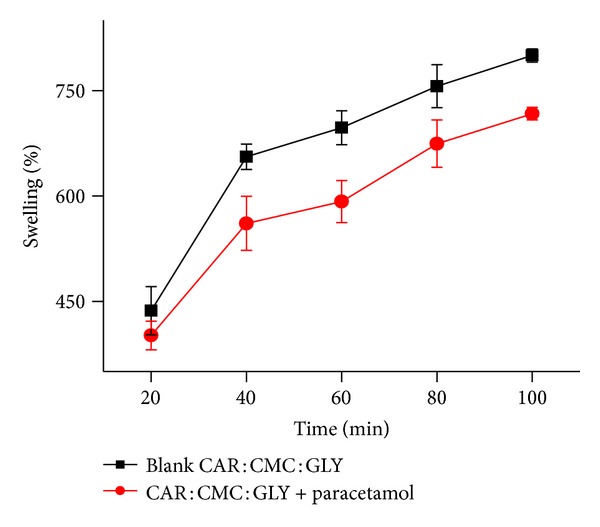
Hydration profile showing the % swelling for the blank and paracetamol loaded film containing CAR : CMC : GLY in phosphate buffer (mean ± s.d, *n* = 3).

Figure 4 shows representative swelling results corresponding to the blank or paracetamol loaded CAR : CMC : GLY films and indicates a gradual increase in percent swelling, respectively, achieving 780% and 730% of the initial weight in 120 minutes. However, the highest rate of swelling occurred within the first 40-minutes as the % swelling reached approximately 680% after which the rate of swelling significantly decreased. The results confirm the capability of the polymeric matrix to swell in PBS solution within a 40-minute period, which is expected to allow subsequent rapid drug release. Similar studies for the blank SA : CMC : GLY or amoxicillin loaded films showed percent swelling of 350% and 390%, respectively, in the first 20 minutes. However, the films started dissolving in the PBS and therefore not expected to maintain its physical structure on mucosal tissues for a long period compared to the CAR : CMC : GLY films. Such differences can have implications for the duration of drug release as well as determine whether release will be rapid or sustained [21].

### 3.8. Mucoadhesion Studies

Table 3 shows the mucoadhesion (stickiness, work of adhesion, and cohesiveness) results for the blank films containing CAR : CMC : GLY or SA : CMC : GLY as well as the corresponding drug loaded films. 

The stickiness is defined as the maximum force (N) required to detach the film from the surface of the agar, while cohesiveness is the distance (mm) the film travels to detach from the agar surface. The work of adhesion represents the work done to detach the film from the agar surface and determined from the area under the force-distance plot [22]. The comparison between the blank films showed similar stickiness and work of adhesion for both formulations, whilst the cohesiveness values for blank CAR : CMC : GLY film were higher but the difference was not significant (*P* = 0.127). The same trend was observed for stickiness and cohesiveness values for paracetamol loaded CMC : CAR : GLY and amoxicillin loaded SA : CMC : GLY films, while the work of adhesion showed a significant difference (*P* = 0.016) between these two films. Mucoadhesive performance is important as it determines the residence time of formulations at the absorption site to allow for sustained drug release and ultimately bioavailability. The overall results confirm that both films showed acceptable mucoadhesive properties which can be attributed to the formation of hydrogen bonds between the CMC, SA, or CAR and agar surface which make them suitable drug delivery carriers for buccal mucosa administration. This is based on the adsorption theory of mucoadhesion which proposes that the presence of intermolecular forces such as hydrogen bonding serves as adhesive interaction between the substrate surfaces [23, 24]. Hydrogen bondformation between polymeric functional groups such as hydroxyl groups and the mucosal surface has been reported for cellulosic polymers such as CMC and CAR [25, 26]. 

### 3.9. *In Vitro* Drug Dissolution Studies

UV visible spectroscopy was used to obtain the linear calibration curves for paracetamol (*R* ^2^ = 0.999) and amoxicillin (*R* ^2^ = 0.998) at wavelengths of 242 and 272 nm, respectively. Drug release experiments were carried out on paracetamol and amoxicillin films and the data fitted to the Korsmeyer-Peppas equation [27]. 

**Figure 5:**
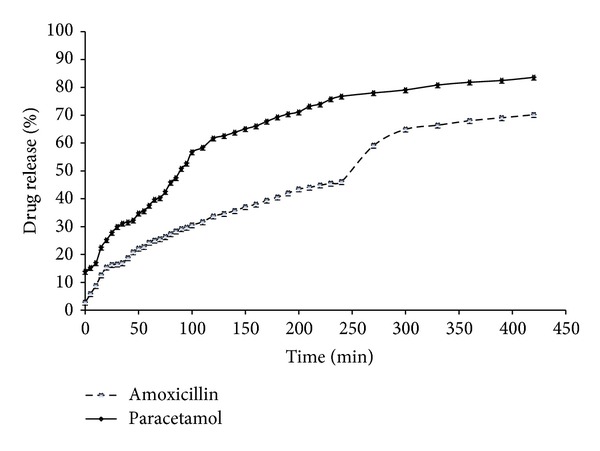
*In vitro *drug dissolution profiles of paracetamol released from films containing CAR : CMC : GLY (1 : 2 : 3) and amoxicillin released from films containing SA : CMC : GLY (5 : 3 : 6).

As shown in Figure 5, the films showed sustained release of the drug with a total cumulative release of 84.65% and 70.59%, respectively, for paracetamol and amoxicillin. The rate of paracetamol (1.88% min^−*n*^) release was faster compared to the amoxicillin (1.04% min^−*n*^) which could be due to the fact that there was excess paracetamol present on the surface of the film with maximum drug loading compared to the maximum amoxicillin loaded films. This difference is however, expected to be more prominent in the initial stages of drug release and not throughout the duration of release, as other mechanisms may be at play. The lower rate of amoxicillin release could also be due to the relatively higher amount of total polymer content SA : CMC : GLY (5 : 3 : 6) and is expected to form a stronger gel which will retard hydration and subsequent drug release by diffusion. Further, the paracetamol loaded films contained CAR whilst the amoxicillin loaded films contained CAR both of which hydrate and swell to different extents and could therefore affect the overall rates of drug release. Drug release from swellable matrices is usually complex, and though some processes may be distinctly classified as either diffusion or erosion controlled, drug release is mostly governed by both mechanisms. Analysis of the experimental data using this equation and interpretation of the release exponents (*n*) provide a better understanding of the mechanisms controlling release. The release exponent (*n*) values were 0.45 and 0.52 for paracetamol and amoxicillin, respectively. These values of *n* show an anomalous (non-Fickian) transport and suggest that diffusion of both drugs through the hydrated polymeric gels combined with gel erosion controlled drug release overall [27]. Similar observations were made for films prepared from only CMC containing paracetamol [19]. It should be noted also that different polymers were used and in different ratios in the optimised films for the two drugs, which makes the dissolution data not directly comparable. 

## 4. Conclusions 

Polymers such as CMC, CAR, and SA together with GLY can be used for increasing the drug loading of amoxicillin and paracetamol both of which are water soluble. This method has promise of providing an ideal buccal mucosal drug delivery system in the future. The maximum loading for both drugs was achieved at 40% (paracetamol) and 26.3% (amoxicillin), and this was possible using combinations of CAR : CMC : GLY (paracetamol) and SA : CMC : GLY (amoxicillin). The two formulations showed differences in adhesive and hydration properties which are expected to influence their mucoadhesive performance on the buccal surface but this requires further investigations.

## Figures and Tables

**Table:** (a) Blank films

Mixture of polymers and plasticizer	Weight ratios	Total polymer weight (mg)
	5 : 3 : 3	1100
SA : CMC : GLY	5 : 3 : 6	1400
	5 : 2 : 3	1000
CAR : CMC : SA : GLY	2 : 2 : 2 : 3	900
CAR : SA : GLY	2 : 3 : 6	1100
CAR : CMC : GLY	1 : 2 : 3	600

**Table:** (b) Drug loaded films

Formulation	Weight without drug (mg)	Weight of drug (mg)	Total dry weight (mg)	Drug loading (% of film dry weight)
Paracetamol				

SA : CMC : GLY (5 : 3 : 3)	1100	100	1200	8.3
SA : CMC : GLY (5 : 3 : 3)	1100	150	1250	12.0
SA : CMC : GLY (5 : 2 : 3)	1000	150	1150	13.0
CAR : CMC : SA : GLY (2 : 2 : 2 : 3)	900	50	950	5.3
CAR : CMC : SA : GLY (2 : 2 : 2 : 3)	900	75	975	7.7
CAR : CMC : SA : GLY (2 : 2 : 2 : 3)	900	200	1100	18.2
CAR : CMC : GLY (1 : 2 : 3)	600	250	850	29.4
CAR : CMC : GLY (1 : 2 : 3)	600	300	900	33.3
CAR : CMC : GLY (1 : 2 : 3)^€^	600	400	1000	40.0

Amoxicillin				

SA : CMC : GLY (5 : 3 : 6)	1400	250	1650	15.2
SA : CMC : GLY (5 : 3 : 6)	1400	300	1700	17.6
SA : CMC : GLY (5 : 3 : 6)	1400	350	1750	20.0
SA : CMC : GLY (5 : 3 : 6)	1400	400	1800	22.2
SA : CMC : GLY (5 : 3 : 6)*	1400	500	1900	26.3
CAR : SA : GLY (2 : 3 : 6)	1100	450	1550	29.0
CAR : CMC : GLY (1 : 2 : 3)	600	500	1100	45.5

^€^Optimised paracetamol loaded films selected for further characterization.

*Optimised amoxicillin loaded films selected for further characterisation.

**Table 2:** TGA results showing the % water content in blank and drug loaded film containing optimum amounts of the two model drugs (mean ± SD (*n* = 3)).

Formulation	Water content (%)
CAR : CMC : GLY	8.18 ± 0.6
SA : CMC : GLY	2.44 ± 0.1
CAR : CMC : GLY + paracetamol	4.99 ± 0.5
SA : CMC : GLY + amoxicillin	3.47 ± 0.6

**Table 3:** Mucoadhesion characteristics for film or drug loaded (paracetamol or amoxicillin) films (mean ± SD (*n* = 3)).

Formulation	Stickiness (N)	Work of adhesion (N·mm)	Cohesiveness (mm)
CAR : CMC : GLY	40.38 ± 1.6	44.23 ± 3.9	1.64 ± 0.3
SA : CMC : GLY	38.56 ± 0.6	37.09 ± 6.9	1.18 ± 0.3
CAR : CMC : GLY + paracetamol	39.36 ± 0.8	45.04 ± 4.3	1.39 ± 0.2
SA : CMC : GLY + amoxicillin	38.14 ± 0.5	28.57 ± 5.6	0.94 ± 0.3
